# Racial/Ethnic Differences and Trends in Pathologic Complete Response Following Neoadjuvant Chemotherapy for Breast Cancer

**DOI:** 10.3390/cancers14030534

**Published:** 2022-01-21

**Authors:** Sung Jun Ma, Lucas M. Serra, Brian Yu, Mark K. Farrugia, Austin J. Iovoli, Han Yu, Song Yao, Oluwadamilola T. Oladeru, Anurag K. Singh

**Affiliations:** 1 Department of Radiation Medicine, Roswell Park Comprehensive Cancer Center, 665 Elm Street, Buffalo, NY 14203, USA; SungJun.Ma@RoswellPark.org (S.J.M.); Mark.Farrugia@RoswellPark.org (M.K.F.); Austin.Iovoli@RoswellPark.org (A.J.I.); 2 Jacobs School of Medicine and Biomedical Sciences, University at Buffalo, The State University of New York, 955 Main Street, Buffalo, NY 14203, USA; Lucasser@buffalo.edu (L.M.S.); Byu24@buffalo.edu (B.Y.); 3 Department of Biostatistics and Bioinformatics, Roswell Park Comprehensive Cancer Center, 665 Elm Street, Buffalo, NY 14203, USA; Han.Yu@RoswellPark.org; 4 Department of Cancer Prevention and Control, Roswell Park Comprehensive Cancer Center, 665 Elm Street, Buffalo, NY 14203, USA; Song.Yao@RoswellPark.org; 5 Department of Radiation Oncology, University of Florida, 2000 SW Archer Road, Gainesville, FL 32610, USA; Ooladeru@UFL.edu

**Keywords:** pCR, preoperative chemo, race, ethnicity, NCDB

## Abstract

**Simple Summary:**

Despite improving rates of pathologic complete response (pCR; the absence of invasive cancer at the time of surgery) among patients with breast cancer who underwent chemotherapy prior to surgery, racial and ethnic minority groups were under-represented in clinical trials. Our study used a large cancer registry database in the United States to evaluate the temporal trend of pCR and patterns of pCR and survival outcomes among diverse racial and ethnic groups. It suggested that although pCR rates improved over time for all groups, pCR rates and survival outcomes varied significantly. For instance, compared to non-Hispanic White women, Black women were less likely to have pCR for triple negative and hormone receptor (HR)-negative, human epidermal growth factor receptor 2 (HER2)-positive tumors, but more likely for HR-positive, HER2-negative tumors. Given such heterogeneous outcomes among various racial and ethnic minority groups, further investigations would be warranted to optimize outcomes among such underserved populations.

**Abstract:**

The purpose of this study was to evaluate nationwide trends in pathologic complete response (pCR) and its racial variations for breast cancer. The National Cancer Database was queried for women from 2010 to 2017 with non-metastatic breast cancer who underwent neoadjuvant chemotherapy. The primary endpoints, pCR and overall survival, were evaluated using Cochran-Armitage test, logistic, and Cox regression multivariable analyses. A total of 104,161 women were analyzed. Overall, pCR improved from 2010 to 2017 (15.1% to 27.2%, trend *p* < 0.001). Compared to non-Hispanic White (NHW) women, Hispanic White (HW) women were more likely to have pCR for hormone receptor (HR)-positive, human epidermal growth factor receptor 2 (HER2)-positive tumors (adjusted odds ratio (aOR) 1.29, 95% confidence interval (CI) 1.08–1.53, *p* = 0.005). Black women were less likely to have pCR for HR-HER2+ tumors (aOR 0.81, 95% CI 0.73–0.89, *p* < 0.001) and triple negative (aOR 0.82, 95% CI 0.77–0.87, *p* < 0.001) tumors, but more likely for HR+HER2- tumors (aOR 1.13, 95% CI 1.03–1.24, *p* = 0.009). Among patients who achieved pCR, Asian or Pacific Islander (API) women were associated with better survival (adjusted hazards ratio (aHR) 0.52, 95% CI 0.33–0.82, *p* = 0.005) than NHW women. Despite positive trends in pCR rates, the likelihood of pCR and survival outcomes may be intricately dependent on racial/ethnic groups and tumor receptor subtypes.

## 1. Introduction

The association of pathologic complete response (pCR) following neoadjuvant chemotherapy with improved survival outcomes in breast cancer, particularly for human epidermal growth factor receptor 2 (HER2)-positive and triple negative subtypes, has been previously reported [1,2]. This trend may be in part due to recent advances in systemic therapy regimens including targeted agents which have conferred pCR rates over 60% [3,4]. Given the favorable prognosis of pCR, regulatory agencies support pCR as a valid surrogate endpoint to evaluate the effectiveness of novel therapies [5], and strategies to de-escalate surgery and radiation in select patients are currently being explored [6,7].

Despite these improvements in breast cancer outcomes, racial/ethnic minority groups were associated with worse survival despite receiving standard of care treatments and having comparable socioeconomic status [8,9,10,11]. However, it appears they were either under-represented or not reported in prior prospective trials evaluating neoadjuvant systemic therapies [3,4]. Specific analysis of breast cancer patients of racial and ethnic minority groups for pCR following neoadjuvant therapy and survival outcomes were limited in previous studies due to small sample sizes and short follow-up periods [12,13,14,15]. Given such limitations, the nationwide trend of pCR outside of clinical trials and its association with patients of diverse racial/ethnic background and their survival remains unclear. To address this knowledge gap, we performed an observational cohort study to compare pCR and survival outcomes stratified by racial and ethnic groups.

## 2. Materials and Methods

Our study was approved by Roswell Park Comprehensive Cancer Center institutional (665 Elm St., Buffalo, NY, USA) review board (BDR-131220). Our report follows the Strengthening the Reporting of Observational Studies in Epidemiology (STROBE) reporting guideline.

The National Cancer Database (NCDB) is a nationwide cancer registry database capturing approximately 67% of all newly diagnosed breast cancer in the United States. It is a joint program by the American College of Surgeons and the American Cancer Society. The database was queried for female patients with stage I-III breast cancer, diagnosed between 2010 and 2017, who underwent neoadjuvant chemotherapy. Follow-up was until the end of 2019. Variables of interest included facility type, facility volume, age, race, insurance, income and education levels, type of residence, Charlson–Deyo comorbidity score (CDS), year of diagnosis, histology, tumor grade, clinical T and N staging, hormone receptor (HR) status, surgery type, surgical margin, lymphovascular space invasion (LVSI), radiation therapy, chemotherapy, endocrine therapy, immunotherapy, duration of postoperative inpatient admission, postoperative readmission within 30 days, adjuvant therapy, time interval between diagnosis and the initiation of neoadjuvant chemotherapy, and time interval between the initiation of neoadjuvant chemotherapy and surgery. Education and income levels were estimated based on the 2016 American Community Survey data, measuring the percentage of adults who did not graduate from high school and the median household income adjusted for 2016 inflation, respectively, in each patient’s zip code. They were stratified by the median value of 10.9% and $50,353, respectively. Time intervals among diagnosis, neoadjuvant chemotherapy, and surgery were stratified based on their median values.

Patients were included for analysis if they had non-metastatic breast cancer and underwent neoadjuvant chemotherapy followed by surgery. Patients of male gender, unknown race, unknown clinical T and N staging, metastatic disease, unknown tumor receptor status, unknown receipt of chemotherapy or surgery, and unknown number of days among diagnosis, neoadjuvant chemotherapy, and surgery were excluded. Those diagnosed prior to the year 2010 were excluded as HER2 status was unavailable in the database. All missing values were coded as unknown for analysis. Clinically pertinent variables such as specific comorbidities, performance status, type and duration of systemic therapy, toxicity profile, tumor recurrence, and breast cancer specific mortality were not captured in the database.

The primary endpoints of this study were pCR (defined as the absence of invasive disease (ypT0/isN0) after neoadjuvant therapy) and overall survival (OS; defined as the time interval between diagnosis and the last follow-up or death by any cause). Those diagnosed in 2017 did not have their OS recorded in the database; thus, they were included only in analysis of pCR. Our hypothesis was that pCR rates improved over the years based on recent trials [3,4] and that there were racial and ethnic differences in pCR and OS [8,9,10,11,12,13,14,15].

Cochran–Armitage test was performed to evaluate the trend of pCR over time. Baseline characteristics were compared using Fisher exact test and Mann–Whitney U test. Logistic multivariable analysis (MVA) was performed to evaluate variables associated with pCR. Cox proportional hazard MVA was performed to evaluate the extent of OS benefit of pCR. MVA models were built based on all statistically significant variables from their univariable analysis (UVA) followed by a backward stepwise elimination. Subgroup analyses were performed based on race/ethnicity and tumor receptor status if interaction analyses were statistically significant.

All *p*-values were two-sided and those less than 0.05 were considered statistically significant. Bonferroni correction was used to adjust for three comparisons among racial and ethnic subgroups (non-Hispanic White (NHW) vs. Hispanic White (HW) women, NHW vs. Black women, and NHW vs. Asian or Pacific Islander (API) women). All analyses were performed using R software version 4.0.3 (R Project for Statistical Computing).

## 3. Results

**Figure 1:**
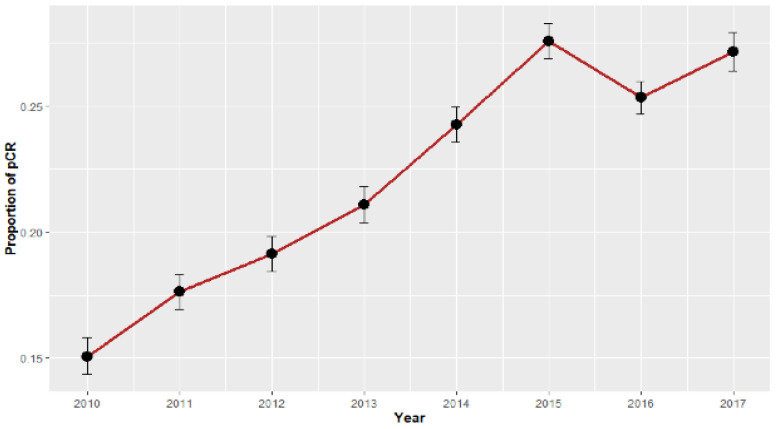
Trends in proportion of pathologic complete response from 2010 to 2017 for all cohorts. Error bar represents 95% confidence interval.

A total of 104,161 patients (n = 72,631 for NHW, n = 7632 for HW, n = 19,505 for Black, n = 4393 for API) met our inclusion criteria (Table S1). The median follow-up was 49.3 months (interquartile range (IQR) 32.8–71.4). The proportion of pCR has increased in recent years for all racial/ethnic groups and tumor receptor subtypes (Figure 1 and Figures S1–S5; all trend *p* < 0.001). Overall pCR rate increased from 15.1% in 2010 to 27.2% in 2017, largely driven by women of API descent (15.7% to 31.6%) and HR-HER2+ tumors (28.6% to 53.1%; all trend *p* < 0.001). Improved pCR rates were also observed in other racial groups (NHW: 14.9% to 27.1%; HW: 16.5% to 30.4%; Black: 15.0% to 24.6%; all trend *p* < 0.001) and other tumor receptor subtypes (HR+HER2-: 6.4% to 11.4%; HR+HER2+: 19.7% to 34.6%; HR-HER2-: 21.3% to 30.4%; all trend *p* < 0.001).

**Figure 2:**
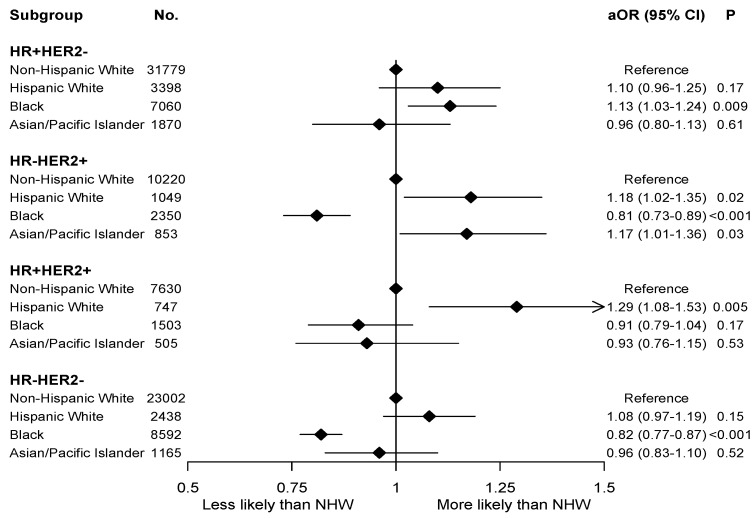
Logistic regression multivariable analysis for pathologic complete response stratified by tumor receptor subtypes and racial groups. No.: number; aOR: adjusted odds ratio; 95% CI: 95% confidence interval; HR: hormone receptor; HER2: human epidermal growth factor receptor 2; NHW: non-Hispanic White.

Logistic MVA was adjusted for age, facility volume, insurance, year of diagnosis, LVSI, facility type, education level, CDS, histology, grade, T and N staging, tumor receptor status, surgery, surgical margin, postoperative readmission, chemotherapy, endocrine therapy, immunotherapy, the use of adjuvant systemic therapy, radiation therapy, and time interval among diagnosis, chemotherapy, and surgery. In this MVA model, compared to NHW women, HW women were more likely to have pCR (adjusted odds ratio (aOR) 1.13, 95% confidence interval (CI) 1.06–1.21, *p* < 0.001), while Black women were less likely to have pCR (aOR 0.90, 95% CI 0.86–0.94, *p* < 0.001). Interaction between race/ethnicity and tumor receptor status was statistically significant (interaction *p* < 0.001). On subgroup analysis (Figure 2), when compared to NHW women, HW women were more likely to have pCR for HR+HER2+ tumors. Black women were less likely to have pCR for HR-HER2+ tumors and triple negative tumors, but more likely for HR+HER2- tumors.

**Figure 3:**
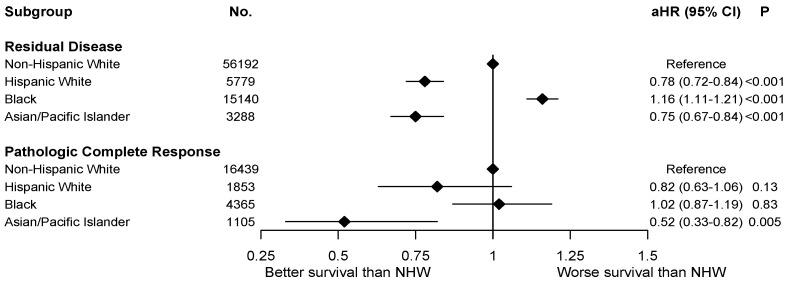
Cox regression multivariable analysis for overall survival stratified by pathologic responses and racial groups. No.: number; aHR: adjusted hazards ratio; 95% CI: 95% confidence interval; NHW: non-Hispanic White.

Cox MVA was adjusted for age, facility volume, insurance, LVSI, facility type, income level, CDS, histology, grade, T and N staging, tumor receptor status, surgery, surgical margin, postoperative readmission, chemotherapy, endocrine therapy, immunotherapy, and time interval between diagnosis and chemotherapy. In this MVA model, pCR was associated with improved OS (adjusted hazards ratio (aHR) 0.31, 95% CI 0.29–0.33, *p* < 0.001). When compared to NHW women, HW (aHR 0.79, 95% CI 0.73–0.85, *p* < 0.001) and API (aHR 0.73, 95% CI 0.65–0.81, *p* < 0.001) women had improved OS, while Black women (aHR 1.15, 95% CI 1.10–1.20, *p* < 0.001) had worse OS. The interaction between race and tumor response was statistically significant (interaction *p* = 0.001), while its interaction with tumor receptor status was not (interaction *p* = 0.053). The three-way interaction among race, tumor receptor status, and tumor response was not statistically significant (interaction *p* = 0.80). On subgroup analysis among patients with residual disease after neoadjuvant chemotherapy, when compared to NHW women, API and HW women were associated with improved OS (Figure 3). Black women were associated with worse OS for residual disease cases only compared to NHW women (Figure 3). Among patients with pCR, only API women remained to be associated with improved OS compared to NHW women (Figure 3).

## 4. Discussion

To our knowledge, this is the largest study using a nationwide oncology database to evaluate the trend of pCR rates over time, the likelihood of achieving pCR for different racial and ethnic groups, and the impact this has on survival stratified by pathologic responses. It suggested that overall pCR rates improved over time, largely due to HER2+ tumors and API women. When compared to NHW women, HW women were more likely to have pCR for HR+HER2+ tumors. Black women were more likely to have pCR for HR+HER2- tumors, but less likely for HR-HER2+ tumors and triple negative tumors. When compared to NHW women, Black women were associated with worse survival for residual tumors without pCR following neoadjuvant systemic therapy. API and HW women were associated with improved survival compared to NHW women for all pathologic responses and any residual disease, respectively.

The trend of improving pCR rates, especially among HER2+ tumors in our study, is consistent with recent trials such as TRAIN-2 that reported rates of nearly 70% in the setting of dual anti-HER2 therapies [4]. Our observation of higher pCR rates with HR-HER2+ tumors compared to HR+HER2+ tumors was also consistent with prior studies [16,17,18,19]. The improvement of pCR rate among triple negative tumors was modest in our study with an increase of 9.1%. The benefit of pembrolizumab for triple negative tumors, including a pCR rate of 65%, was recently reported in 2020 and was likely not reflected in our study as only women diagnosed until the end of 2017 were included for analysis [3]. The improvement in the pCR rate for HR+HER2- tumors was the smallest in our study at 5.0%. Although the NSABP B-40 trial demonstrated the OS and pCR benefit with neoadjuvant bevacizumab, this finding was not observed in other trials [20,21]. In addition, the magnitude of OS benefit of pCR has been shown to be not as significant for HR+HER2- tumors in a recent meta-analysis [2].

Our study suggests several racial differences regarding pCR which serve as opportunities for further investigation and intervention. First, our results suggest that Black women were more likely to have pCR for HR+HER2- tumors and less likely for HR-HER2+ and triple negative tumors. Some studies previously demonstrated comparable pCR regardless of race, while other studies showed that Black women were less likely to have pCR for HER2+ and triple negative tumors, consistent with our study [12,13,14,15,22]. Since select HR-HER2+ tumors may not respond well to anti-HER2 therapies, these tumors biologically may behave like triple negative breast cancers, thus resulting in a lower likelihood of pCR following neoadjuvant chemotherapy [15,23]. In addition, it has been previously reported that Black women undergo suboptimal breast cancer treatments including early discontinuation of systemic therapy or experience treatment delays [9]. Thus, the lower rate of pCR observed among Black women in our study may be in part due to their incomplete courses of neoadjuvant chemotherapy in non-clinical trial settings. This observation may explain comparable pCR rates seen regardless of races among patients enrolled in clinical trials [14]. However, our study also noted a higher pCR rate for HR+HER2- tumors among Black women. It may be in part due to the association of higher 21-gene recurrence score (RS) with a higher likelihood of pCR, suggesting more aggressive tumor biology with higher RS seen in Black women [24,25,26].

Among those without pCR when compared to NHW women, our report suggested that HW women had improved OS and that Black women had worse OS. Prior studies investigating the role of racial differences in survival did not stratify by pathologic responses [8,9,10,11]. However, they reported that Black women had worse survival outcomes, while API and HW women had favorable survival outcomes [8,9,10,11]. Although a single-institution study stratified patients by pCR and different racial groups, there was no statistically significant interaction between them for survival in part due to a short follow-up and small subgroup sample sizes [12]. Our finding on API women with improved OS despite comparable pCR rates compared to NHW women is consistent with reports of a higher level of immune response with tumor-infiltrating lymphocytes among API women, which was associated with improved survival [27,28]. Similarly, our findings in Black women are consistent with a report of having more exhausted CD8+ T cell profiles, which was associated with worse survival [29].

Our primary study limitations were related to the retrospective nature of our dataset. Pertinent variables, such as body mass index, the duration and specific agents of systemic therapy, toxicity profiles, and adherence to systemic therapy, were not captured in the NCDB. While non-White patients were less likely to complete their neoadjuvant therapies in other studies, this finding alone may not fully explain higher pCR rates seen in our study among Black women for HR+HER2- tumors and HW women for HR+HER2+ tumors [9]. Racial disparities in survival outcomes persisted even among patients enrolled in prior clinical trials [8,9,10,11]. In addition, no statistically significant interaction between race and body mass index (BMI) was noted for pathologic response outcomes [14], and non-obese Black women still had worse outcomes [11]. Nonetheless, our study adjusted for patient demographics and treatment characteristics, such as medical insurance, income level, CDS, and potential treatment delays (the duration of interval among diagnosis, the initiation of neoadjuvant chemotherapy, and surgery) when building Cox and logistic MVA models. Even though our MVA models were also adjusted for missing variables, there may be still potential bias associated with them that may affect survival outcomes [30]. Despite such limitations, the strength of our study included being the largest report using a nationwide cancer registry database to evaluate the temporal trend of pCR, patterns of pCR for diverse racial and ethnic groups, and survival outcomes stratified by pathologic responses.

## 5. Conclusions

Despite positive trends in pCR rates, the likelihood of pCR and survival outcomes may be intricately dependent on racial/ethnic groups and tumor receptor subtypes. Further prospective studies are warranted to identify determinants of suboptimal neoadjuvant treatments influencing pCR and appropriately tailor systemic therapies based on tumor biology.

## Data Availability

The primary dataset (National Cancer Database) is available publicly for investigators associated with Commission on Cancer-accredited programs through the American College of Surgeons Available online: https://www.facs.org/quality-programs/cancer/ncdb (accessed on 18 January 2022).

## Supplementary Materials

The following are available online at https://www.mdpi.com/article/10.3390/cancers14030534/s1, Figure S1: Trends in proportion of pathologic complete response from 2010 to 2017 stratified by tumor receptor subtypes; Figure S2: Trends in proportion of pathologic complete response from 2010 to 2017 for non-Hispanic White women; Figure S3: Trends in proportion of pathologic complete response from 2010 to 2017 for Hispanic White women; Figure S4: Trends in proportion of pathologic complete response from 2010 to 2017 for Black women; Figure S5: Trends in proportion of pathologic complete response from 2010 to 2017 for Asian or Pacific Islander women; Table S1: Baseline characteristics of all cohorts stratified by racial groups.

## References

[1] Cortazar P., Zhang L., Untch M., Mehta K., Costantino J.P., Wolmark N., Bonnefoi H., Cameron D., Gianni L., Valagussa P. (2014). Pathological complete response and long-term clinical benefit in breast cancer: The CTNeoBC pooled analysis. Lancet.

[2] Spring L.M., Fell G., Arfe A., Sharma C., Greenup R.A., Reynolds K.L., Smith B.L., Alexander B.M., Moy B., Isakoff S.J. (2020). Pathologic Complete Response after Neoadjuvant Chemotherapy and Impact on Breast Cancer Recurrence and Survival: A Comprehensive Meta-analysis. Clin. Cancer Res..

[3] Schmid P., Cortes J., Pusztai L., McArthur H., Kümmel S., Bergh J., Denkert C., Park Y.H., Hui R., Harbeck N. (2020). Pembrolizumab for Early Triple-Negative Breast Cancer. N. Engl. J. Med..

[4] van Ramshorst M.S., van der Voort A., van Werkhoven E.D., Mandjes I.A., Kemper I., Dezentjé V.O., Oving I.M., Honkoop A.H., Tick L.W., van de Wouw A.J. (2018). Neoadjuvant chemotherapy with or without anthracyclines in the presence of dual HER2 blockade for HER2-positive breast cancer (TRAIN-2): A multicentre, open-label, randomised, phase 3 trial. Lancet Oncol..

[5] Food and Drug Administration (2014). Guidance for Industry: Pathological Complete Response in Neoadjuvant Treatment of High- Risk Early-Stage Breast Cancer: Use as an Endpoint to Support Accelerated Approval.

[6] Sutton E.J., Braunstein L.Z., El-Tamer M.B., Brogi E., Hughes M., Bryce Y., Gluskin J.S., Powell S., Woosley A., Tadros A. (2021). Accuracy of Magnetic Resonance Imaging–Guided Biopsy to Verify Breast Cancer Pathologic Complete Response After Neoadjuvant Chemotherapy. JAMA Netw. Open.

[7] Mamounas E.P., Bandos H., White J.R., Julian T.B., Khan A.J., Shaitelman S.F., Torres M.A., Vicini F., Ganz P.A., McCloskey S.A. (2019). NRG Oncology/NSABP B-51/RTOG 1304: Phase III trial to determine if chest wall and regional nodal radiotherapy (CWRNRT) post mastectomy (Mx) or the addition of RNRT to whole breast RT post breast-conserving surgery (BCS) reduces invasive breast cancer recurrence-free interval (IBCR-FI) in patients (pts) with pathologically positive axillary (PPAx) nodes who are ypN0 after neoadjuvant chemotherapy (NC). J. Clin. Oncol..

[8] Albain K.S., Unger J.M., Crowley J.J., Coltman C.A., Hershman D.L. (2009). Racial Disparities in Cancer Survival Among Randomized Clinical Trials Patients of the Southwest Oncology Group. JNCI J. Natl. Cancer Inst..

[9] Hershman D.L., Unger J.M., Barlow W.E., Hutchins L.F., Martino S., Osborne C.K., Livingston R.B., Albain K.S. (2009). Treatment Quality and Outcomes of African American Versus White Breast Cancer Patients: Retrospective Analysis of Southwest Oncology Studies S8814/S8897. J. Clin. Oncol..

[10] Newman L.A., Griffith K.A., Jatoi I., Simon M.S., Crowe J.P., Colditz G. (2006). Meta-Analysis of Survival in African American and White American Patients With Breast Cancer: Ethnicity Compared With Socioeconomic Status. J. Clin. Oncol..

[11] Sparano J.A., Wang M., Zhao F., Stearns V., Martino S., Ligibel J.A., Perez E.A., Saphner T., Wolff A., Sledge G.W. (2012). Race and Hormone Receptor–Positive Breast Cancer Outcomes in a Randomized Chemotherapy Trial. JNCI J. Natl. Cancer Inst..

[12] Mac Gregor M.C., Litton J.K., Chen H., Giordano S.H., Hudis C.A., Wolff A., Valero V., Hortobagyi G.N., Bondy M.L., Gonzalez-Angulo A.M. (2010). Pathologic complete response in breast cancer patients receiving anthracycline- and taxane-based neoadjuvant chemotherapy. Cancer.

[13] Dawood S., Broglio K., Kau S.-W., Green M.C., Giordano S.H., Meric-Bernstam F., Buchholz T.A., Albarracin C., Yang W.T., Hennessy B.T. (2009). Triple Receptor–Negative Breast Cancer: The Effect of Race on Response to Primary Systemic Treatment and Survival Outcomes. J. Clin. Oncol..

[14] Warner E.T., Ballman K.V., Strand C., Boughey J.C., Buzdar A.U., Carey L.A., Sikov W.M., Partridge A.H. (2016). Impact of race, ethnicity, and BMI on achievement of pathologic complete response following neoadjuvant chemotherapy for breast cancer: A pooled analysis of four prospective Alliance clinical trials (A151426). Breast Cancer Res. Treat..

[15] Killelea B.K., Yang V.Q., Wang S.-Y., Hayse B., Mougalian S., Horowitz N.R., Chagpar A.B., Pusztai L., Lannin D.R. (2015). Racial Differences in the Use and Outcome of Neoadjuvant Chemotherapy for Breast Cancer: Results From the National Cancer Data Base. J. Clin. Oncol..

[16] Gianni L., Pienkowski T., Im Y.-H., Roman L., Tseng L.-M., Liu M.-C., Lluch A., Staroslawska E., De La Haba-Rodriguez J., Im S.-A. (2012). Efficacy and safety of neoadjuvant pertuzumab and trastuzumab in women with locally advanced, inflammatory, or early HER2-positive breast cancer (NeoSphere): A randomised multicentre, open-label, phase 2 trial. Lancet Oncol..

[17] Moon H.-G., Im S.-A., Han W., Oh D.-Y., Han S.-W., Keam B., Park I.A., Chang J.M., Moon W.K., Cho N. (2012). Estrogen receptor status confers a distinct pattern of response to neoadjuvant chemotherapy: Implications for optimal durations of therapy: Distinct patterns of response according to ER expression. Breast Cancer Res. Treat..

[18] Ring A., Smith I., Ashley S., Fulford L.G., Lakhani S.R. (2004). Oestrogen receptor status, pathological complete response and prognosis in patients receiving neoadjuvant chemotherapy for early breast cancer. Br. J. Cancer.

[19] Zambetti M., Mansutti M., Gómez P., Lluch A., Dittrich C., Zamagni C., Ciruelos E., Pavesi L., Semiglazov V., De Benedictis E. (2011). Pathological complete response rates following different neoadjuvant chemotherapy regimens for operable breast cancer according to ER status, in two parallel, randomized phase II trials with an adaptive study design (ECTO II). Breast Cancer Res. Treat..

[20] Bear H.D., Tang G., Rastogi P., Geyer C.E., Robidoux A., Atkins J.N., Baez-Diaz L., Brufsky A.M., Mehta R.S., Fehrenbacher L. (2012). Bevacizumab Added to Neoadjuvant Chemotherapy for Breast Cancer. N. Engl. J. Med..

[21] Nahleh Z., Botrus G., Dwivedi A., Jennings M., Nagy S., Tfayli A. (2019). Bevacizumab in the neoadjuvant treatment of human epidermal growth factor receptor 2-negative breast cancer: A meta-analysis of randomized controlled trials. Mol. Clin. Oncol..

[22] Meng M., Wang H., Zaorsky N.G., Sun B., Zhu L., Song Y., Li F., Dong Y., Wang J., Chen H. (2019). Risk-adapted stereotactic body radiation therapy for central and ultra-central early-stage inoperable non-small cell lung cancer. Cancer Sci..

[23] Singer C.F., Tan Y., Fitzal F., Steger G.G., Egle D., Reiner A., Rudas M., Moinfar F., Gruber C., Petru E. (2017). Pathological Complete Response to Neoadjuvant Trastuzumab Is Dependent on HER2/CEP17 Ratio in HER2-Amplified Early Breast Cancer. Clin. Cancer Res..

[24] Gianni L., Zambetti M., Clark K., Baker J., Cronin M., Wu J., Mariani G., Rodriguez J., Carcangiu M., Watson D. (2005). Gene Expression Profiles in Paraffin-Embedded Core Biopsy Tissue Predict Response to Chemotherapy in Women With Locally Advanced Breast Cancer. J. Clin. Oncol..

[25] Holowatyj A.N., Cote M.L., Ruterbusch J.J., Ghanem K., Schwartz A.G., Vigneau F.D., Gorski D.H., Purrington K.S. (2018). Racial Differences in 21-Gene Recurrence Scores Among Patients With Hormone Receptor–Positive, Node-Negative Breast Cancer. J. Clin. Oncol..

[26] Hoskins K.F., Danciu O.C., Ko N.Y., Calip G.S. (2021). Association of Race/Ethnicity and the 21-Gene Recurrence Score With Breast Cancer–Specific Mortality Among US Women. JAMA Oncol..

[27] Kan Z., Ding Y., Kim J., Jung H.H., Chung W., Lal S., Cho S., Fernandez-Banet J., Lee S.K., Kim S.W. (2018). Multi-omics profiling of younger Asian breast cancers reveals distinctive molecular signatures. Nat. Commun..

[28] Pan J.W., Zabidi M.M.A., Ng P.-S., Meng M.-Y., Hasan S.N., Sandey B., Sammut S.-J., Yip C.-H., Rajadurai P., Rueda O.M. (2020). The molecular landscape of Asian breast cancers reveals clinically relevant population-specific differences. Nat. Commun..

[29] Yao S., Cheng T.-Y.D., Elkhanany A., Yan L., Omilian A., I Abrams S., Evans S., Hong C.-C., Qi Q., Davis W. (2021). Breast Tumor Microenvironment in Black Women: A Distinct Signature of CD8+ T-Cell Exhaustion. JNCI: J. Natl. Cancer Inst..

[30] Yang D.X., Khera R., Miccio J.A., Jairam V., Chang E., Yu J.B., Park H.S., Krumholz H.M., Aneja S. (2021). Prevalence of Missing Data in the National Cancer Database and Association With Overall Survival. JAMA Netw. Open.

