# Supplementary material for: Racial/Ethnic Differences and Trends in Pathologic Complete Response Following Neoadjuvant Chemotherapy for Breast Cancer
Source: Cancers (Basel). 2022 Jan 21;14(3):534. doi: 10.3390/cancers14030534 (PMC8833599; doi:10.3390/cancers14030534)
Supplement: Supplementary file 1 [file cancers-14-00534-s001.zip › cancers-1511503-supplementary.pdf]

Table S1. Baseline characteristics of all cohorts stratified by racial groups.

	NHW		HW		Black		API		
Variable	N	%	N	%	N	%	N	%	P
pCR									<0.001
No	56192	77.4	5779	75.7	15140	77.6	3288	74.8	
Yes	16439	22.6	1853	24.3	4365	22.4	1105	25.2	
Total	72631	100.0	7632	100.0	19505	100.0	4393	100.0	
Facility									<0.001
Nonacademic	43333	59.7	3517	46.1	9751	50.0	2038	46.4	
Academic	19913	27.4	2534	33.2	6873	35.2	1521	34.6	
Not available	9385	12.9	1581	20.7	2881	14.8	834	19.0	
Total	72631	100.0	7632	100.0	19505	100.0	4393	100.0	
Facility volume									<0.001
Low	4697	6.5	360	4.7	776	4.0	243	5.5	
Int	14355	19.8	1446	18.9	3329	17.1	804	18.3	
High	53579	73.8	5826	76.3	15400	79.0	3346	76.2	
Total	72631	100.0	7632	100.0	19505	100.0	4393	100.0	
Age									<0.001
<50	26753	36.8	4075	53.4	8058	41.3	2166	49.3	
50-70	38821	53.4	3155	41.3	10125	51.9	1976	45.0	
>70	7057	9.7	402	5.3	1322	6.8	251	5.7	
Total	72631	100.0	7632	100.0	19505	100.0	4393	100.0	
Insurance									<0.001
None	1460	2.0	1050	13.8	917	4.7	197	4.5	
Private	49013	67.5	3675	48.2	10892	55.8	3003	68.4	
Government	21410	29.5	2813	36.9	7454	38.2	1140	26.0	
Not available	748	1.0	94	1.2	242	1.2	53	1.2	
Total	72631	100.0	7632	100.0	19505	100.0	4393	100.0	
Income									<0.001
Above median	42155	58.0	3410	44.7	6561	33.6	3080	70.1	
Below median	20552	28.3	3274	42.9	10207	52.3	743	16.9	
Not available	9924	13.7	948	12.4	2737	14.0	570	13.0	

Total	72631	100.0	7632	100.0	19505	100.0	4393	100.0	
Education									<0.001
Educated	39336	54.2	1831	24.0	5459	28.0	2261	51.5	
Uneducated	23481	32.3	4863	63.7	11327	58.1	1564	35.6	
Not available	9814	13.5	938	12.3	2719	13.9	568	12.9	
Total	72631	100.0	7632	100.0	19505	100.0	4393	100.0	
Residence									<0.001
Metro	60167	82.8	7206	94.4	17790	91.2	4171	94.9	
Urban	9301	12.8	307	4.0	1155	5.9	114	2.6	
Rural	1184	1.6	17	0.2	158	0.8	5	0.1	
Not available	1979	2.7	102	1.3	402	2.1	103	2.3	
Total	72631	100.0	7632	100.0	19505	100.0	4393	100.0	
CDS									<0.001
0	64103	88.3	6693	87.7	16089	82.5	3932	89.5	
1	6888	9.5	803	10.5	2689	13.8	397	9.0	
2+	1640	2.3	136	1.8	727	3.7	64	1.5	
Total	72631	100.0	7632	100.0	19505	100.0	4393	100.0	
Year of diagnosis									<0.001
2010-2013	31250	43.0	3130	41.0	8226	42.2	1685	38.4	
2014-2017	41381	57.0	4502	59.0	11279	57.8	2708	61.6	
Total	72631	100.0	7632	100.0	19505	100.0	4393	100.0	
Histology									<0.001
Ductal	57526	79.2	6305	82.6	16210	83.1	3632	82.7	
Lobular	4617	6.4	334	4.4	657	3.4	140	3.2	
Others	10488	14.4	993	13.0	2638	13.5	621	14.1	
Total	72631	100.0	7632	100.0	19505	100.0	4393	100.0	
Grade									<0.001
Well diff	3908	5.4	350	4.6	589	3.0	180	4.1	
Moderately diff	22941	31.6	2236	29.3	4518	23.2	1328	30.2	
Poorly diff	40582	55.9	4514	59.1	13055	66.9	2568	58.5	
Others	214	0.3	24	0.3	67	0.3	17	0.4	
Not available	4986	6.9	508	6.7	1276	6.5	300	6.8	

Total	72631	100.0	7632	100.0	19505	100.0	4393	100.0	
cT									<0.001
1	14019	19.3	1184	15.5	3430	17.6	634	14.4	
2	38261	52.7	4099	53.7	10065	51.6	2537	57.8	
3	14618	20.1	1777	23.3	4280	21.9	877	20.0	
4	5733	7.9	572	7.5	1730	8.9	345	7.9	
Total	72631	100.0	7632	100.0	19505	100.0	4393	100.0	
cN									<0.001
0	33836	46.6	3141	41.2	7733	39.6	1972	44.9	
1	30748	42.3	3430	44.9	8963	46.0	1943	44.2	
2	4974	6.8	642	8.4	1716	8.8	284	6.5	
3	3073	4.2	419	5.5	1093	5.6	194	4.4	
Total	72631	100.0	7632	100.0	19505	100.0	4393	100.0	
HR									<0.001
HR+/HER2-	31779	43.8	3398	44.5	7060	36.2	1870	42.6	
HR-/HER2+	10220	14.1	1049	13.7	2350	12.0	853	19.4	
HR+/HER2+	7630	10.5	747	9.8	1503	7.7	505	11.5	
HR-/HER2-	23002	31.7	2438	31.9	8592	44.1	1165	26.5	
Total	72631	100.0	7632	100.0	19505	100.0	4393	100.0	
Surgery									<0.001
Lumpectomy	26002	35.8	2684	35.2	8031	41.2	1571	35.8	
Mastectomy	46571	64.1	4943	64.8	11465	58.8	2818	64.1	
Others	58	0.1	5	0.1	9	0.0	4	0.1	
Total	72631	100.0	7632	100.0	19505	100.0	4393	100.0	
Margin									0.40
Negative	68701	94.6	7182	94.1	18424	94.5	4148	94.4	
Positive	3007	4.1	335	4.4	829	4.3	196	4.5	
Not available	923	1.3	115	1.5	252	1.3	49	1.1	
Total	72631	100.0	7632	100.0	19505	100.0	4393	100.0	
LVSI									<0.001
No	38889	53.5	3934	51.5	10183	52.2	2287	52.1	
Yes	15346	21.1	1663	21.8	3781	19.4	907	20.6	
Not available	18396	25.3	2035	26.7	5541	28.4	1199	27.3	

Total	72631	100.0	7632	100.0	19505	100.0	4393	100.0	
RT									<0.001
No	21084	29.0	2303	30.2	5045	25.9	1226	27.9	
Yes	49156	67.7	5032	65.9	13697	70.2	3030	69.0	
Not available	2391	3.3	297	3.9	763	3.9	137	3.1	
Total	72631	100.0	7632	100.0	19505	100.0	4393	100.0	
Chemo									<0.001
Multi-agent	68530	94.4	7068	92.6	18504	94.9	4149	94.4	
Others	4101	5.6	564	7.4	1001	5.1	244	5.6	
Total	72631	100.0	7632	100.0	19505	100.0	4393	100.0	
Endocrine therapy									<0.001
No	37937	52.2	4051	53.1	12110	62.1	2269	51.7	
Yes	33204	45.7	3403	44.6	6990	35.8	2020	46.0	
Not available	1490	2.1	178	2.3	405	2.1	104	2.4	
Total	72631	100.0	7632	100.0	19505	100.0	4393	100.0	
Immunotherapy									<0.001
No	62636	86.2	6603	86.5	17230	88.3	3577	81.4	
Yes	9842	13.6	996	13.1	2213	11.3	800	18.2	
Not available	153	0.2	33	0.4	62	0.3	16	0.4	
Total	72631	100.0	7632	100.0	19505	100.0	4393	100.0	
Postoperative inpatient duration									<0.001
0-1 days	51008	70.2	5154	67.5	13094	67.1	3222	73.3	
>1 day	12236	16.8	1431	18.8	3643	18.7	755	17.2	
Not available	9387	12.9	1047	13.7	2768	14.2	416	9.5	
Total	72631	100.0	7632	100.0	19505	100.0	4393	100.0	
Postoperative readmission within 30 days									<0.001
None	69664	95.9	7243	94.9	18656	95.6	4224	96.2	
Unplanned	796	1.1	149	2.0	233	1.2	44	1.0	
Planned	823	1.1	132	1.7	235	1.2	47	1.1	
Others	50	0.1	10	0.1	15	0.1	4	0.1	
Not available	1298	1.8	98	1.3	366	1.9	74	1.7	
Total	72631	100.0	7632	100.0	19505	100.0	4393	100.0	

Adjuvant therapy									<0.001
No	42938	59.1	4574	59.9	12786	65.6	2455	55.9	
Yes	29693	40.9	3058	40.1	6719	34.4	1938	44.1	
Total	72631	100.0	7632	100.0	19505	100.0	4393	100.0	
Time between dx and NACT									<0.001
0-30 days	37910	52.2	2724	35.7	7345	37.7	1998	45.5	
>30 days	34721	47.8	4908	64.3	12160	62.3	2395	54.5	
Total	72631	100.0	7632	100.0	19505	100.0	4393	100.0	
Time between NACT and surgery									<0.001
0-150 days	36432	50.2	3246	42.5	8715	44.7	2078	47.3	
>150 days	36199	49.8	4386	57.5	10790	55.3	2315	52.7	
Total	72631	100.0	7632	100.0	19505	100.0	4393	100.0	

NHW: non-Hispanic White; HW: Hispanic White; API: Asian and Pacific Islander; N: number; pCR: pathologic complete response; Int: intermediate; CDS: Charlson-Deyo comorbidity score; diff: differentiated; HR: hormone receptor; HER2: human epidermal growth factor receptor 2; LVSI: lymphovascular space invasion; RT: radiation therapy; Chemo: chemotherapy; dx: diagnosis; NACT: neoadjuvant chemotherapy.

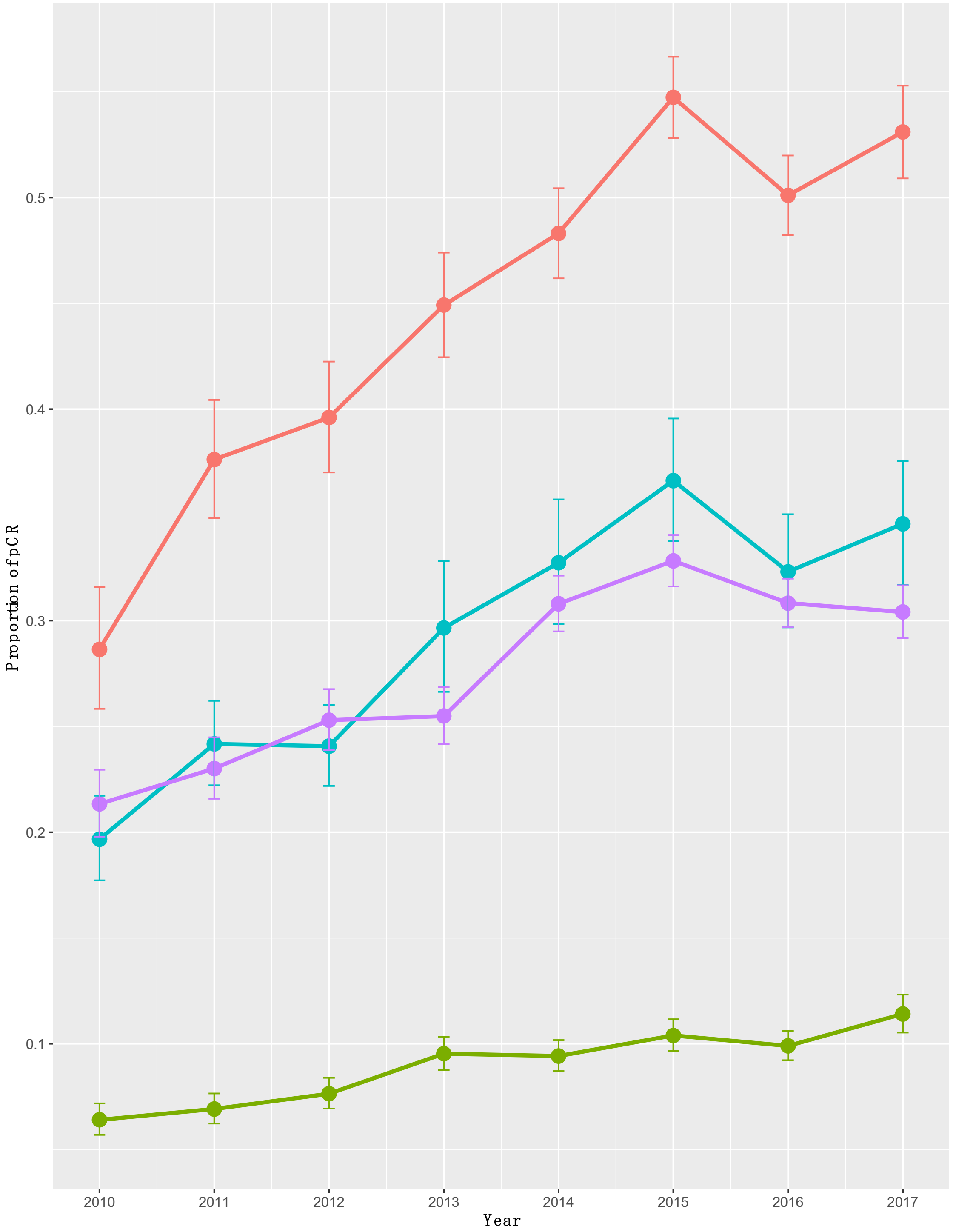

Figure S1. Trends in proportion of pathologic complete response from 2010 to 2017 stratified by tumor receptor subtypes. Orange: HR-HER2+; cyan: HR+HER2+; purple: HR-HER2-; green: HR+HER2-. Error bar represents 95% confidence interval. pCR: pathologic complete response; HR: hormone receptor; HER2: human epidermal growth factor receptor 2.

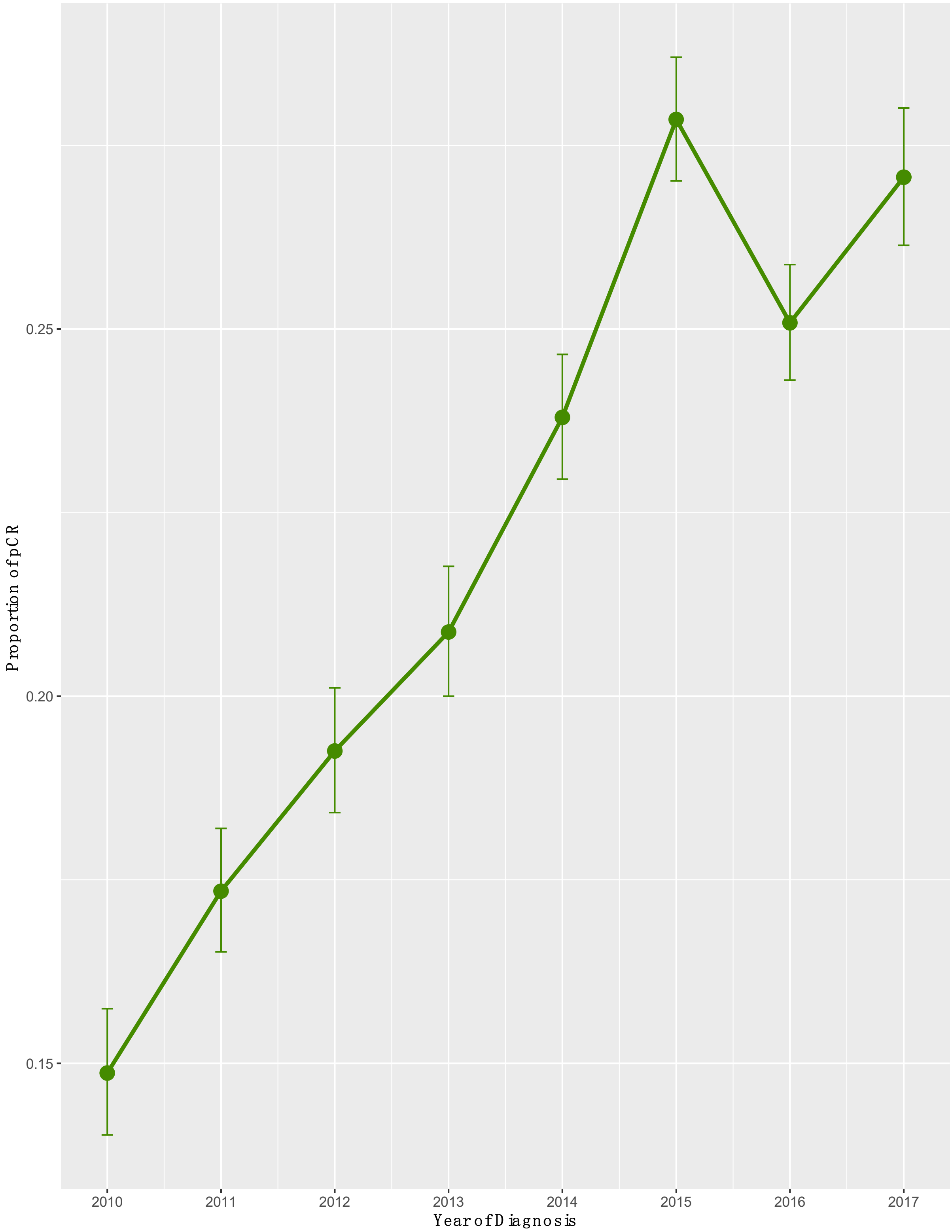

Figure S2. Trends in proportion of pathologic complete response from 2010 to 2017 for non-Hispanic White women. Error bar represents 95% confidence interval.

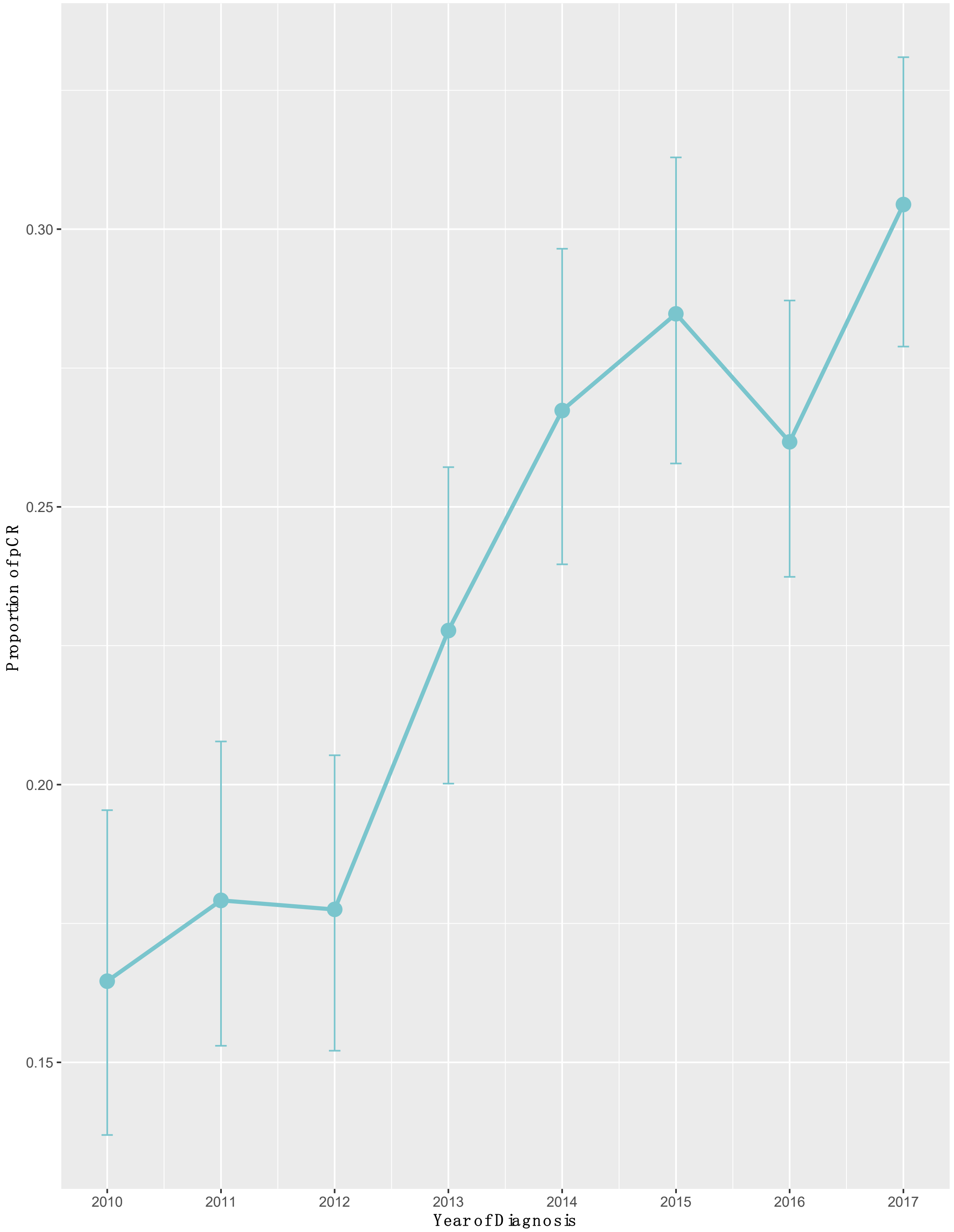

Figure S3. Trends in proportion of pathologic complete response from 2010 to 2017 for Hispanic White women. Error bar represents 95% confidence interval.

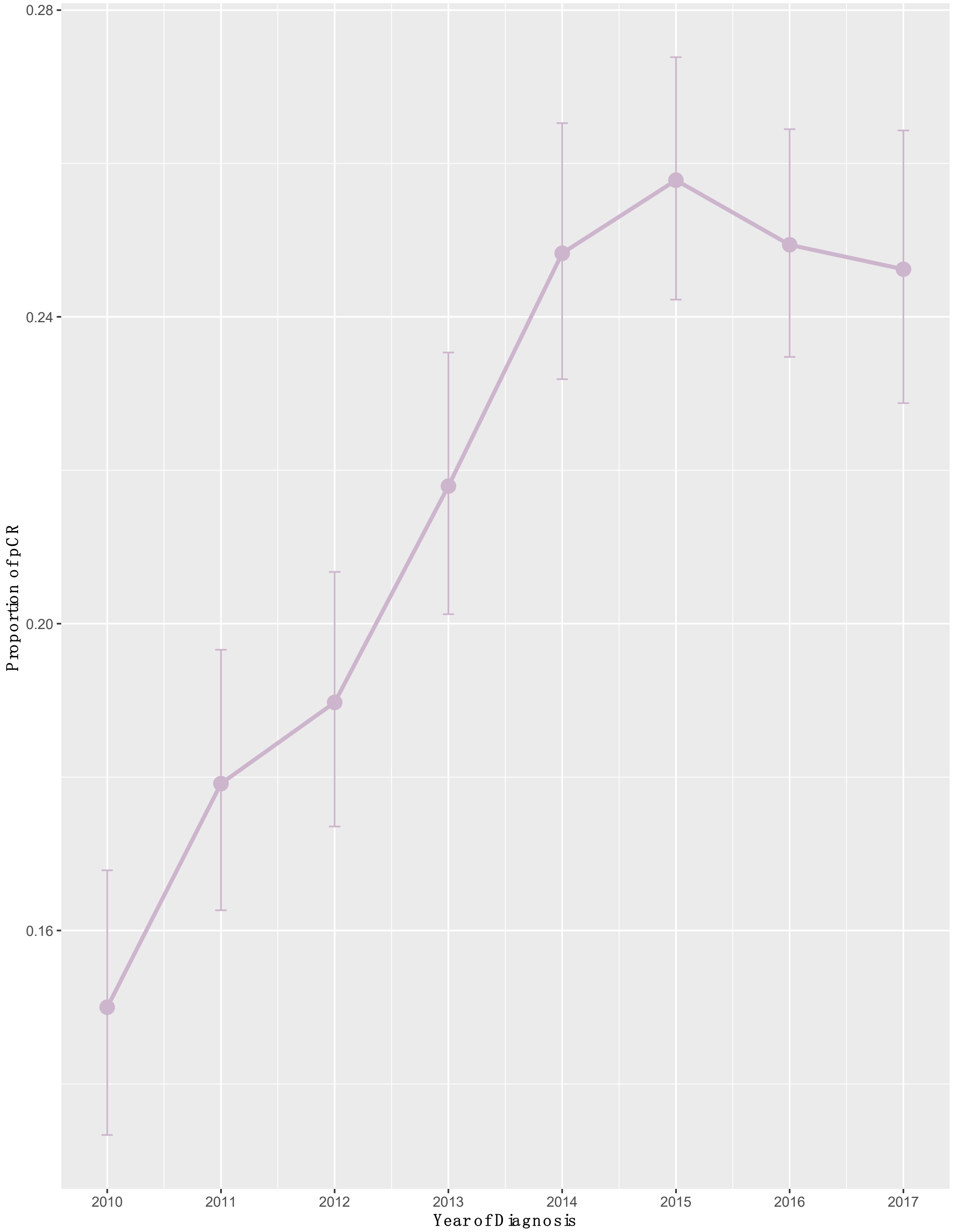

Figure S4. Trends in proportion of pathologic complete response from 2010 to 2017 for Black women. Error bar represents 95% confidence interval.

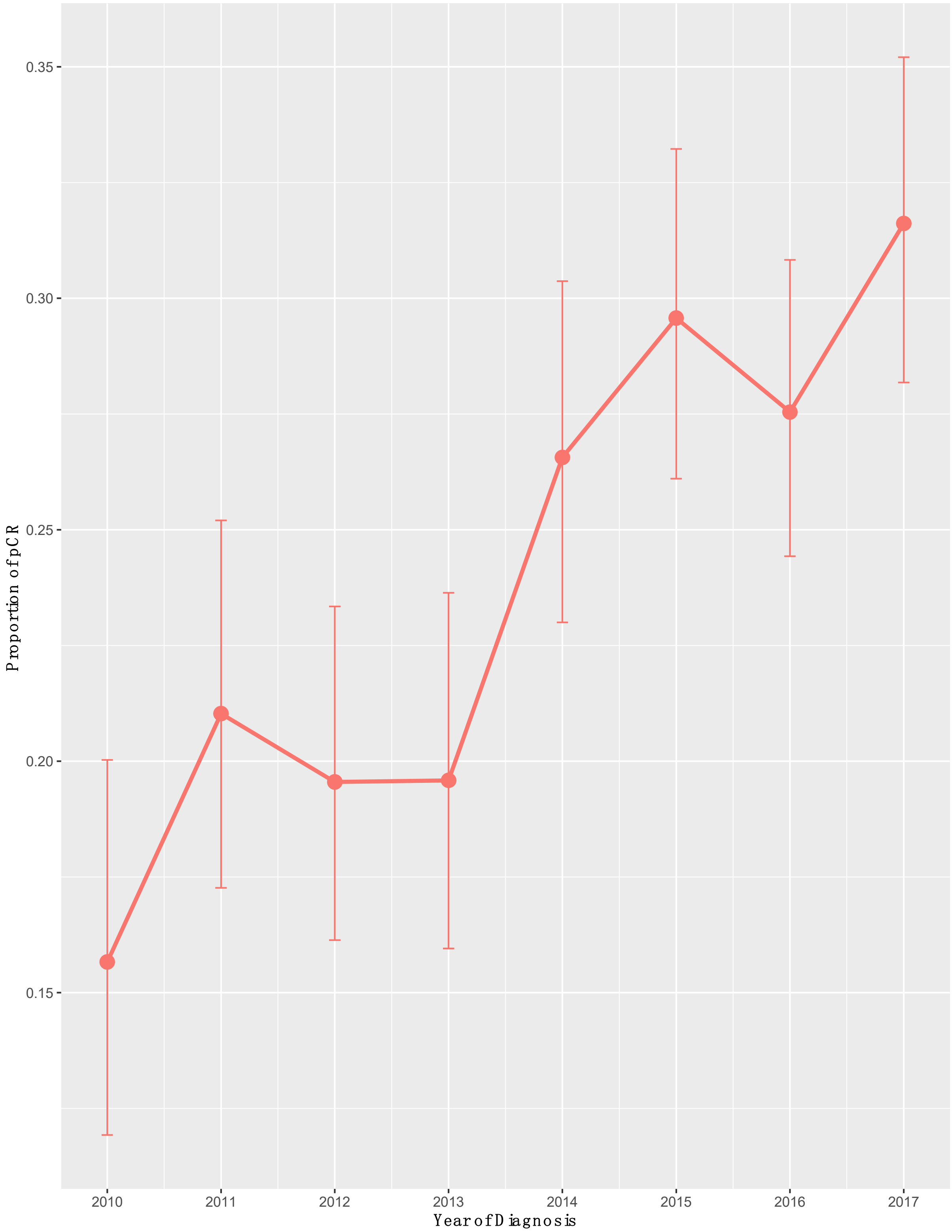

Figure S5. Trends in proportion of pathologic complete response from 2010 to 2017 for Asian or Pacific Islander women. Error bar represents 95% confidence interval.
